# Impact of the clinical role of interventional radiologists: results of the CLINTERVENTIONAL randomized controlled trial

**DOI:** 10.1007/s00330-025-11757-0

**Published:** 2025-06-17

**Authors:** Pedro Blas García Jurado, Juan José Espejo Herrero, María Sagrario Lombardo Galera, María Eugenia Pérez Montilla, Sara Barranco Acosta, José García-Revillo García, Pilar Font Ugalde, Marina Álvarez Benito

**Affiliations:** 1 Maimónides Biomedical Research Institute of Córdoba (IMIBIC), Córdoba, Spain; 2 University of Córdoba, Córdoba, Spain; 3 Department of Radiology, Reina Sofía University Hospital, Córdoba, Spain

**Keywords:** Audiovisual Aids, Communication, Patient satisfaction, Physician–patient relations, Radiology (interventional)

## Abstract

**Objectives:**

To assess the impact of preprocedural consultations with interventional radiologists and explanatory videos of interventional radiology (IR) procedures on patients’ knowledge, satisfaction with information and communication, and anxiety regarding the procedure.

**Materials and methods:**

A randomized, controlled, single-center trial (ClinicalTrials.gov: NCT05461482) was conducted between August 2022 and April 2024. Patients scheduled for certain IR procedures were included. They were randomly assigned to a control group (standard information from the ordering physician) or experimental group (additional consultation with an interventional radiologist and access to explanatory videos about the procedures). Knowledge of the procedures (measured via multiple-choice questionnaires), satisfaction with the information and communication (Likert scales), and anxiety (Likert scale and State-Trait Anxiety Inventory) were assessed. Statistical analyses included Student’s *t*-test, the chi-square test, and mixed analysis of variance.

**Results:**

Four hundred thirty patients (mean age, 62 years (13); 267 men) were included. The experimental group (*n* = 214), compared to the control group (*n* = 216), showed greater understanding of the procedures (10.5 (1.9) vs 5.1 (3.2); *p* < 0.001) and greater satisfaction with the information (8.9 (1.5) vs 6.5 (3.3); *p* < 0.001) and communication (8.7 (1.7) vs 6.4 (2.8); *p* < 0.001). Anxiety was lower in the experimental group according to the State-Trait Anxiety Inventory (42.9 (12.7) vs 45.7 (12.4); *p* = 0.02). 99.5% (207/208) of patients in the experimental group felt the video helped them understand the intervention.

**Conclusions:**

Preprocedural consultations by interventional radiologists improve patients’ understanding of the procedure, increase their satisfaction with information and communication, and reduce anxiety during the procedure.

**Key Points:**

***Question*** *Technical advances in IR lack parallel clinical role development, possibly limiting preprocedural information. This study evaluates preprocedural consultations and videos to address this gap*.

***Findings*** *Preprocedural consultations with interventional radiologists and explanatory videos enhance patient knowledge, improve satisfaction with information and communication, and reduce procedure-related anxiety*.

***Clinical relevance*** *Structured preprocedural communication, including consultations with radiologists and explanatory videos, optimizes patients’ understanding, satisfaction with information and communication, and emotional well-being, reinforcing the importance of patient-centered care in IR*.

**Graphical Abstract:**

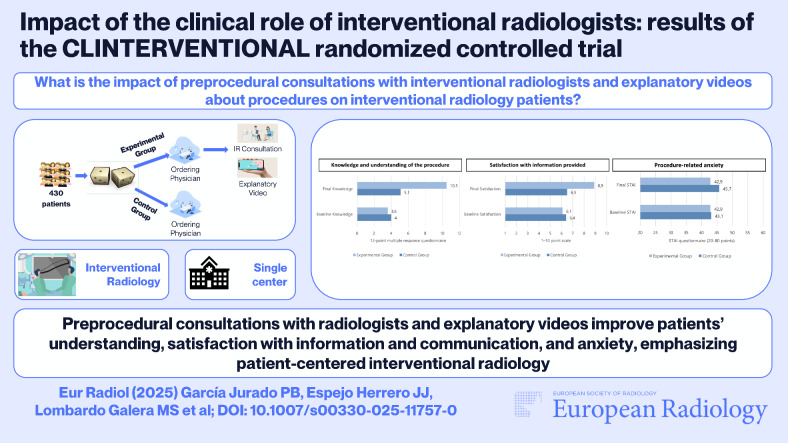

## Introduction

Technical advances in interventional radiology (IR) have positioned this discipline as a fundamental specialty in modern medicine, thanks to its minimally invasive techniques for managing a wide variety of complex diseases [1, 2]. However, the clinical development of IR, especially in terms of taking direct responsibility for the patient and longitudinal patient care, has not advanced at the same pace [3–5]. This gap generates significant challenges in the doctor–patient relationship, highlighting the need for a more integrative approach that improves communication and strengthens the interaction between radiologists and patients. Moving toward value-based, patient-centered radiology [6, 7] could generate very significant benefits, such as a stronger radiologist–patient relationship, a more satisfying patient experience, the promotion of research as a result of better follow-up, and, ultimately, greater visibility of IR [8, 9].

Scientific societies and IR experts agree that in order to consolidate the specialty definitively, it is crucial for interventional radiologists to take on a more active clinical role [1, 10, 11]. However, published data on the impact of consultations with interventional radiologists on patients attended to by IR departments are limited [8, 12, 13]. This highlights the need for work with robust methods on patient-centered care in IR. In this context, the integration of audiovisual tools for educational purposes has emerged as a promising strategy whose effectiveness has already been demonstrated in other clinical settings [14–16].

This randomized clinical trial hypothesizes that the implementation of preprocedural consultations by IR specialists, along with the use of explanatory videos on procedures, improves patient understanding, increases satisfaction with the information provided, and reduces procedure-related anxiety. This study seeks to generate data that will contribute to advancing toward an IR that is closer to and more focused on a patient’s needs, as indicated in current recommendations.

## Materials and methods

The CLINTERVENTIONAL trial, a prospective study registered at clinicaltrials.gov (NCT05461482), was approved by the Córdoba Research Ethics Committee. Participants received an information sheet and signed the informed consent form (Appendix 1). The trial was conducted in accordance with the Declaration of Helsinki.

### Study design and objectives

**Fig. 1:**
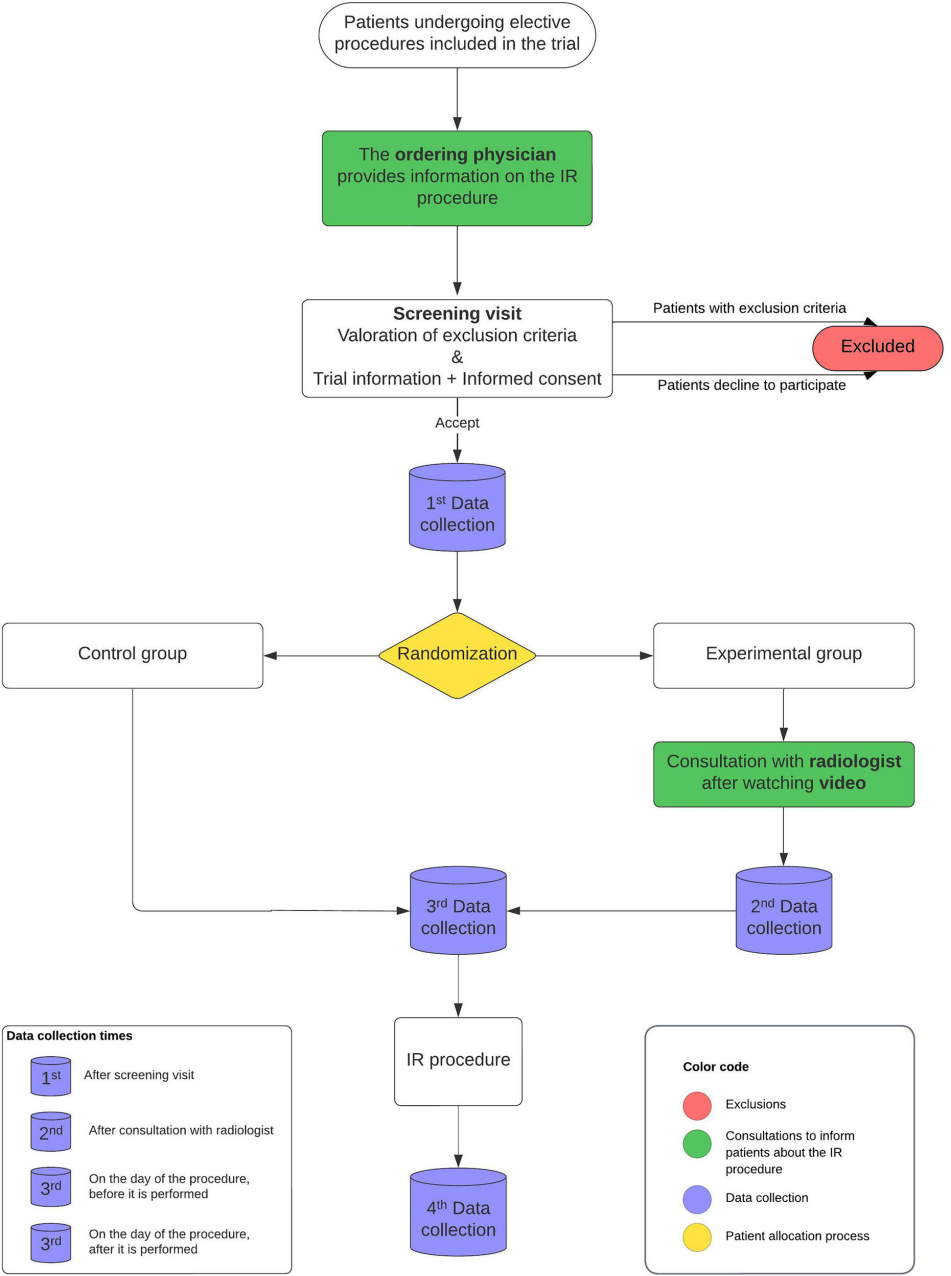
Flowchart illustrating the study design, showing a schematic summary of critical time points throughout the trial. IR, interventional radiology

A randomized, controlled, single-center, two-arm clinical trial was conducted at the Reina Sofía University Hospital (Córdoba, Spain) between 8/31/2022 and 4/17/2024. Participants undergoing the following procedures were selected: endovascular recanalizations, placement of tunneled central venous catheters, fistulography and treatment of hemodialysis fistulas, embolizations, biopsies, drainage of collections, transhepatic biliary drainage, and nephrostomies. Participants were randomly assigned (1:1) by an independent coordinator to the control or experimental group using random number tables (Fig. 1). In the control group, participants received information on the IR procedure from the ordering physician, following the usual hospital protocol. In the experimental group, participants received an information leaflet with a Quick Response code that linked to an animated video explaining the procedure (Appendix 2). Eight videos were created—one for each procedure included—with an approximate duration of 4 min. They also had an additional consultation with an interventional radiologist (PBGJ, MSLG, MEPM, and SBA with 6, 11, 9, and 4 years of experience, respectively), who explained the procedure and answered questions.

The videos, created using animation to avoid overly realistic images, shared a common structure with six sections: introduction, procedure description, benefits, preparation, risks, and expectations after the procedure. The scripts were developed based on informed consent forms and patients’ most frequently asked questions. They were subsequently reviewed by individuals without a medical background to ensure the language was accessible and appropriate for patients.

Participants, data analysts, and outcome evaluators were blinded.

The main objective was to compare both groups at the baseline, corresponding to the first visit, and at the end of the study, on the day of the IR procedure, in terms of participants’ knowledge of the IR procedure, satisfaction with the information provided and method of communication, and anxiety.

The secondary objectives were to analyze intragroup changes in the same variables as the main objective between baseline and the end of the study; compare the duration of the intervention, pain during the procedure, level of tolerance of the intervention, and level of satisfaction with the intervention between both groups; evaluate the explanatory videos as an educational tool; and analyze the association of the different variables studied with pain during the intervention, tolerance of the procedure, and satisfaction with the intervention.

This paper will analyze the main objective and the first three secondary objectives.

### Participants

Consecutive patients scheduled for the aforementioned elective interventions were included. Those under 18 years of age, those who required assistance for consent, pregnant women, those who did not speak Spanish, those with allergies to the drugs used, and those with sensory impairments were excluded.

### Outcome measures and data collection

Knowledge about the interventions was assessed with eight multiple-choice questionnaires that were specifically designed and validated using a systematic approach and pilot testing (Appendix 3). The other main variables (satisfaction with the information, satisfaction with the method of communication, and anxiety) were assessed via 10-point visual analog scales. In addition, anxiety was assessed using the State-Trait Anxiety Inventory (STAI).

Demographic data; personal medical history; variables related to the procedure, pain tolerance, and satisfaction with the intervention; and usefulness of the videos were also collected (Table 1).

**Table 1:** Variables, measurement method, and data collection times

Variables	Measurement method	Data collection time^a^			
**Main variables**					
Knowledge and understanding of the procedure	12-point specific multiple-response questionnaire for each procedure (see Appendix 3)^b^	1st	2nd	3rd	
Satisfaction with the method of communicating information	10-point VAS^c^	1st	2nd	3rd	
Satisfaction with information transmitted	10-point VAS^c^	1st	2nd	3rd	
Anxiety related to the procedure	STAI questionnaire and 10-point VAS^c^	1st	2nd	3rd	
**Other variables**					
Demographic variables	Sex, age, marital status, and educational level	1st			
Personal history	Previous IR procedures, other types of interventions, history of anxiety and depression, and previous knowledge of IR	1st			
Procedure-specific variables	Preparation method, anesthesia type, sedation level (Ramsay Sedation Scale), and procedure duration				4th
Pain intensity during the procedure	10-point VAS^d^				4th
Intervention tolerance level	10-point VAS^c^				4th
Satisfaction level with the intervention	10-point VAS^c^				4th
Usefulness of explanatory videos	10-point VAS^c^ and dichotomous questions^e^		2nd		

*IR* interventional radiology, *STAI* State-Trait Anxiety Inventory, *VAS* visual analog scale

^a^ Data collection times: 1st (screening visit), 2nd (after the consultation with the radiologist, only for the experimental group), 3rd (on the day of the procedure, before it is performed), and 4th (on the day of the procedure, after it is performed)

^b^ Scoring system: 1 point for each correct answer

^c^ VAS, where 1 represents the minimum value and 10 the maximum value

^d^ VAS for pain, where 0 indicates no pain, 1–3 mild pain, 4–6 moderate pain, 7–8 severe pain, and 9–10 excruciating pain

^e^ The questions were: Did you find the video useful for better understanding the intervention? Did you like the video as an explanatory tool? Do you feel less anxious after watching the video? Do you understand the intervention better after watching the video?

Data were collected at four points in time: (1) screening visit, (2) after consultation with the radiologist (experimental group only), (3) on the day of the procedure before the procedure, and (4) on the day of the procedure after the procedure (Table 1).

The data were anonymized, collected in digital and paper format, and stored in a database by external clinical care coordinators.

### Statistical analysis

A descriptive study was conducted, calculating absolute and relative frequencies for qualitative variables and means and standard deviations (SDs) for quantitative variables. Confidence intervals were calculated with a confidence level of 95%.

Student’s *t*-test was used for independent data to compare quantitative variables between the control and experimental groups. The chi-square test was used to compare qualitative variables. In addition, a mixed analysis of variance with the Šidák correction was performed to analyze the main objective and first secondary objective, simultaneously comparing the intragroup and intergroup variability of the main variables.

All comparisons were two-tailed and considered significant when *p* < 0.05. The analysis was performed with SPSS® Version 29.0 (IBM).

The sample size was calculated using the GRANMO Calculator. Based on a study by Lattuca et al [17], a difference of at least 1 was assumed in the knowledge and comprehension score, with a SD of 3.5. Therefore, the estimated effect size (Cohen’s *d*) was 0.285. In addition, a bilateral design, an alpha risk of 0.05, a beta risk of 0.2, a 1:1 allocation ratio, and a loss-to-follow-up rate of 10% were established. In the end, 214 subjects per group were needed.

The statistical analysts were PBGJ and PFU.

## Results

### Patient characteristics

**Fig. 2:**
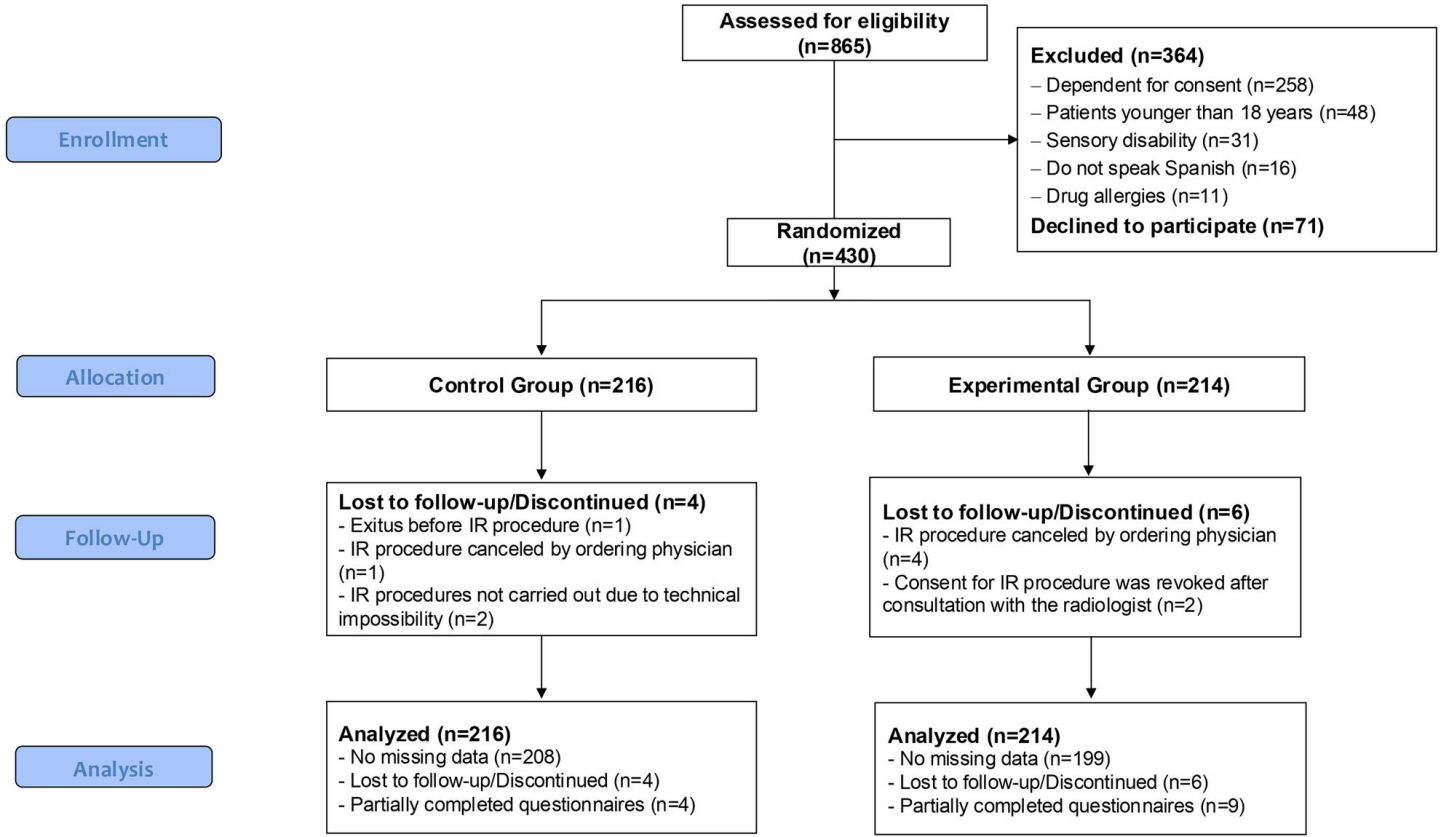
Study the flow chart. The diagram shows the process from eligibility assessment to inclusion in the final analysis. IR, interventional radiology

Of the 865 patients electively scheduled for the interventions included in the study during the study period, 364 were excluded: 258 were dependent for granting informed consent, 48 were under 18 years of age, 31 had sensory impairments, 16 did not speak Spanish, and 11 had drug allergies to the medications used. In addition, 71 declined to participate. The final cohort included 430 participants: 216 assigned to the control group and 214 to the experimental group. All participants were included in the analysis, although 10 participants discontinued the study for different reasons and 13 only partially completed the questionnaires (Fig. 2). The sample included 267 men (62.1%) and 163 women (37.9%) with a mean age of 62.2 (13.6) years. No evidence was found of differences in the baseline characteristics between groups, listed in Table 2.

**Table 2:** Demographic and clinical characteristics of patients in the control and experimental groups

Variables	Control (*n* = 216)	Experimental (*n* = 214)	*p*^a^
Age (years)^b^	62.1 (13.8)	62.3 (13.5)	0.73
Sex, *n* (%)			
Men	141 (65.3)	126 (58.9)	0.17
Women	75 (34.7)	88 (41.1)	
Education level, *n* (%)			
None	27 (12.5)	34 (15.9)	
Primary	76 (35.2)	64 (29.9)	
Secondary	43 (19.9)	37 (17.3)	0.25
Baccalaureate	15 (6.9)	23 (10.7)	
Vocational training	32 (14.8)	24 (11.2)	
University	23 (10.6)	32 (15)	
Marital status, *n* (%)			
Single	26 (12)	26 (12.1)	
Married	150 (69.4)	154 (72)	0.85
Divorced	20 (9.3)	15 (7)	
Widowed	20 (9.3)	19 (8.9)	
History of anxiety/depression, *n* (%)	71 (32.9)	81 (37.9)	0.28
Knowledge of the existence of the IR, *n* (%)	27 (12.5)	33 (15.4)	0.38
Prior interventions, *n* (%)	168 (77.8)	168 (78.5)	0.86
Prior IR interventions, *n* (%)	21 (9.7)	26 (12.1)	0.42
Admission type, *n* (%)			
Hospitalized	114 (52.8)	115 (53.7)	0.84
Outpatient clinic	102 (47.2)	99 (46.3)	
Type of intervention, *n* (%)			
Biopsy	78 (36.1)	79 (36.9)	
Nephrostomy	38 (17.6)	38 (17.8)	
Transhepatic biliary drainage	19 (8.8)	19 (8.9)	
Collection drainage	39 (18.1)	45 (21)	0.93
Tunneled central venous catheter	29 (13.4)	23 (10.7)	
Embolization	5 (2.3)	2 (0.9)	
Recanalization	1 (0.5)	1 (0.5)	
Fistulography	7 (3.2)	7 (3.3)	

*IR* interventional radiology

^a^ Statistical significance based on Student’s *t*-test (quantitative variables) and the chi-square test (qualitative variables)

^b^ Continuous variable expressed as mean and SD

### Understanding of the interventions, satisfaction with information and communication, and anxiety related to interventions

**Fig. 3:**
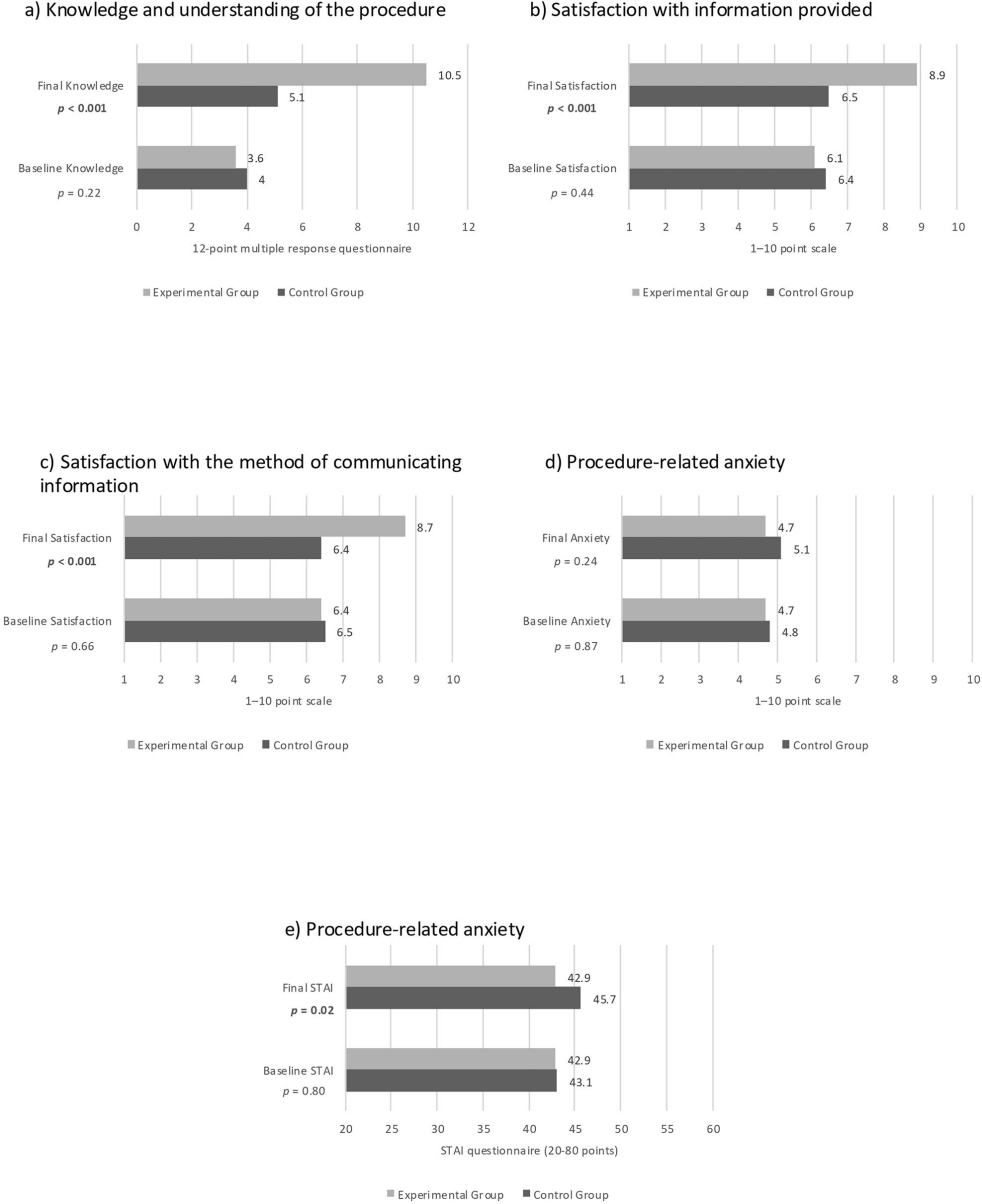
Results of the comparison between groups of the main variables at baseline and at the end of the study: **a** Patients’ knowledge and understanding of the procedures. **b** Satisfaction with the information provided. **c** Satisfaction with the method of communication. **d** Procedure-related anxiety measured on a scale of 1–10. **e** Procedure-related anxiety evaluated with the STAI

At baseline, no evidence was found of a difference between groups in knowledge and understanding of the procedures (*p* = 0.22), satisfaction with the information (*p* = 0.44), satisfaction with the method of communication (*p* = 0.66), and anxiety according to a 1–10 scale (*p* = 0.87) and the STAI (*p* = 0.80). On the contrary, on the day of the procedure, the experimental group showed greater understanding of the interventions (10.5 (1.9) vs 5.1 (3.2); *p* < 0.001), greater satisfaction with the information (8.9 (1.5) vs 6.5 (3.3); *p* < 0.001) and method of communication (8.7 (1.7) vs 6.4 (2.8); *p* < 0.001), and less anxiety according to the STAI questionnaire (42.9 (12.7) vs 45.7 (12.4); *p* = 0.02) compared to the control group (Table 3 and Fig. 3).

**Table 3:** Analysis of the variability of the main variables between and within groups at baseline and at the end of the study

Variables (mean and SD)		Control	Experimental	*p*^a^
Understanding of interventions (0–12)	Baseline	4 (2.8)	3.6 (3)	0.22
	Final	5.1 (3.2)^b^	10.5 (1.9)^b^	< 0.001
Satisfaction with information (1–10)	Baseline	6.4 (2.9)	6.1 (3)	0.44
	Final	6.5 (3.3)^c^	8.9 (1.5)^b^	< 0.001
Satisfaction with communication method (1–10)	Baseline	6.5 (2.9)	6.4 (2.9)	0.66
	Final	6.4 (2.8)^c^	8.7 (1.7)^b^	< 0.001
Anxiety (1–10)	Baseline	4.8 (3.1)	4.7 (3.1)	0.87
	Final	5.1 (2.9)^c^	4.7 (2.9)^c^	0.24
STAI anxiety questionnaire (20–80)	Baseline	43.1 (11)	42.9 (10.9)	0.80
	Final	45.7 (12.4)^b^	42.9 (12.7)^c^	0.02

*SD* standard deviation, *STAI* State-Trait Anxiety Inventory

^a^ Statistical significance between groups based on mixed analysis of variance

^b^ Intragroup statistical significance *p* < 0.001

^c^ Intragroup statistical significance *p* > 0.05

**Fig. 4:**
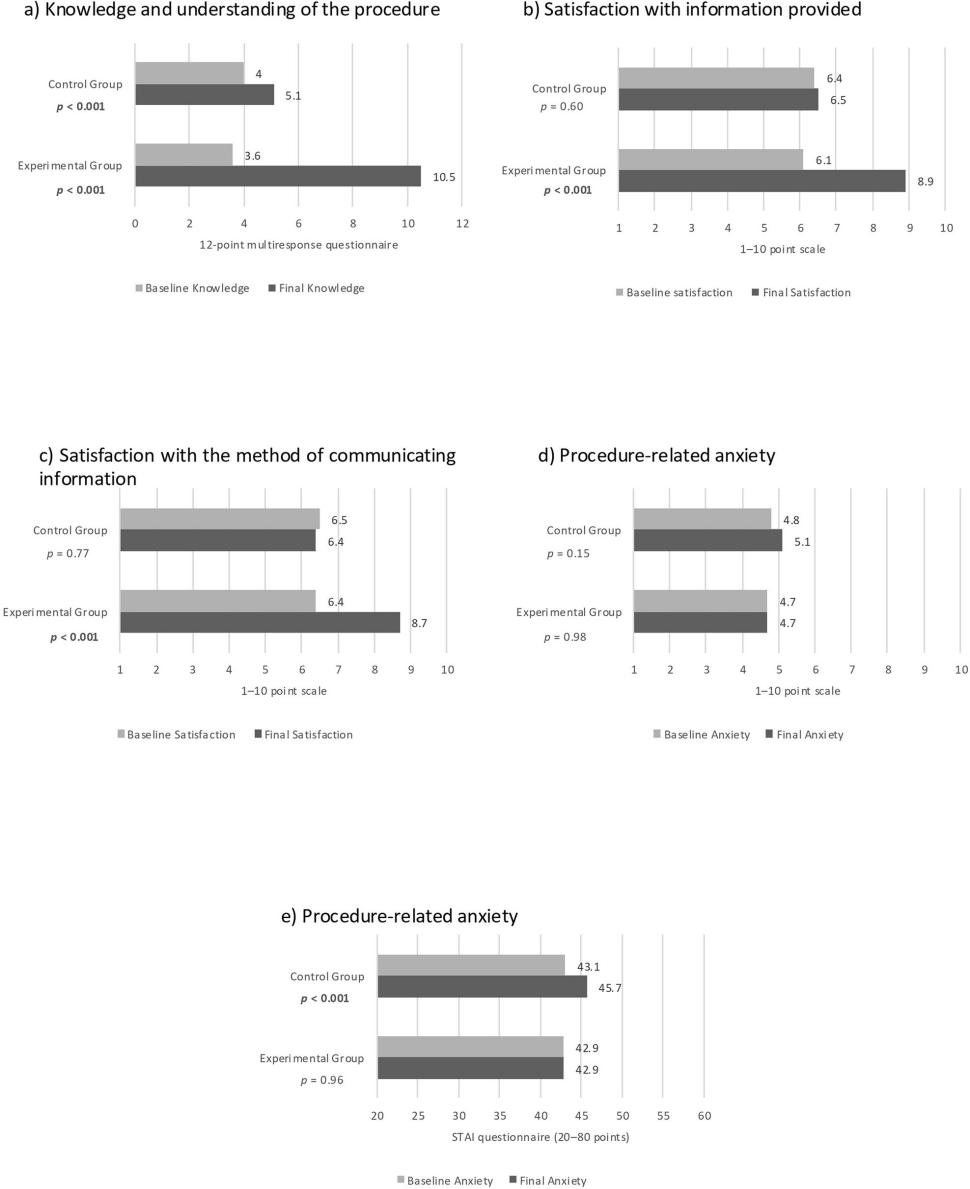
Results of the intragroup comparison of the main variables at baseline and at the end of the study: **a** Patients’ knowledge of the procedures. **b** Satisfaction with the information provided. **c** Satisfaction with the method of communication. **d** Anxiety measured on a scale of 1–10. **e** Anxiety evaluated with the STAI

Regarding intragroup variability, understanding improved in both groups from the beginning to the end of the study (the control group went from 4 (2.8) to 5.1 (3.2), *p* < 0.001; and the experimental group went from 3.6 (3) to 10.5 (1.9); *p* < 0.001). However, satisfaction with the information and communication method increased only in the experimental group (satisfaction with information increased from 6.1 (3) to 8.9 (1.5); *p* < 0.001; and satisfaction with communication method increased from 6.4 (2.9) to 8.7 (1.7); *p* < 0.001), while anxiety measured via the STAI questionnaire increased only in the control group (from 43.1 (11) to 45.7 (12.4); *p* < 0.001) (Table 3 and Fig. 4).

### Duration, pain, tolerance, and satisfaction with the interventions

Regarding intervention-related variables measured on the same day at the end of the intervention, no evidence was found of differences between the control and experimental groups in terms of procedure duration (33.6 (18) vs 33.5 (19.4); *p* = 0.94), pain experienced (2.6 (2.5) vs 2.7 (2.7); *p* = 0.93), tolerance of the intervention (8.6 (1.8) vs 8.8 (1.7); *p* = 0.10), and satisfaction with the intervention (9.2 (1.2) vs 9.2 (1.5); *p* = 0.14) (Table 4).

**Table 4:** Comparative analysis of variables related to the evaluation of interventions

Variables	Group	Mean and SD	*p*^a^
Duration of intervention (min)	Control	33.6 (18)	0.94
	Experimental	33.5 (19.4)	
Pain during the intervention (scale of 1–10)	Control	2.6 (2.5)	0.93
	Experimental	2.7 (2.7)	
Tolerance of the intervention (scale of 1–10)	Control	8.6 (1.8)	0.10
	Experimental	8.8 (1.7)	
Satisfaction with the intervention (scale of 1–10)	Control	9.2 (1.2)	0.14
	Experimental	9.2 (1.5)	

*SD* standard deviation

^a^ Statistical significance based on Student’s *t*-test

### Videos as an educational tool

Of the 208 participants in the experimental group who viewed the explanatory videos and completed the corresponding questionnaire, 99.5% (207/208) considered that the video helped them better understand the intervention, while 99% (206/208) rated it as a useful tool for explaining the procedure. In addition, 86.5% (180/208) reported feeling less anxiety regarding the intervention after watching the video. In regard to the evaluation of the videos on a scale of 1 to 10, participants gave a mean score of 9.4 (1.2), highlighting their usefulness in understanding the procedure, its benefits, the preparation required, and the associated risks.

## Discussion

Technical advances in IR have not been accompanied by an equivalent progression in its clinical role. This affects the doctor–patient relationship and hinders the consolidation of this discipline. This work presents the results of a randomized clinical trial evaluating the impact of preprocedural consultations with interventional radiologists and explanatory videos on the interventions. Participants who received the additional consultation and viewed the video had a better understanding of the interventions (10.5 (1.9) vs 5.1 (3.2); *p* < 0.001) and were more satisfied with the information received (8.9 (1.5) vs 6.5 (3.3); *p* < 0.001) and the method of communication (8.7 (1.7) vs 6.4 (2.8); *p* < 0.001). In addition, they had less anxiety according to the specific STAI questionnaire (42.9 (12.7) vs 45.7 (12.4); *p* = 0.02). The measures did not influence pain (2.6 (2.5) vs 2.7 (2.7); *p* = 0.93), tolerance (8.6 (1.8) vs 8.8 (1.7); *p* = 0.10), or satisfaction with the intervention (9.2 (1.2) vs 9.2 (1.5); *p* = 0.14). The majority felt the videos helped to improve understanding (99.5% (207/208)) and reduce anxiety 86.5% (180/208), supporting their role as an educational tool in IR.

Despite the recommendations of European and American societies and international consensuses [1, 10, 11], quantitative evidence on the impact of consultations on patients’ knowledge and perception remains limited [8, 12, 13, 18]. Culverwell et al reported better understanding in patients informed by radiologists vs surgeons (19/22 (86%) vs 35/66 (53%); *p* = 0.001), although there was no difference in anxiety (radiologists 3.75 vs surgeons 4.59; *p* = 0.26) [13]. Lutjeboer et al also reported greater satisfaction in patients seen in consultations by radiologists (2.68 (0.31) vs 2.48 (0.38); *p* < 0.001), highlighting the dimension of information and communication, which showed an improvement of 0.26 points (*p* < 0.001) [12].

There is a high level of evidence on the impact of videos on the cognitive and emotional sphere, although the existing studies do not focus on IR [14–16, 19–24]. The effectiveness of audiovisual tools in improving the understanding of medical procedures is well documented [15, 19–22], especially in interventions with feedback [16], like this trial. However, its effect on anxiety is controversial. Most studies do not identify clear advantages [14, 19, 21, 23], although Monteiro et al suggest these tools may reduce it [24]. In this trial, the differences consisted of stable anxiety in the experimental group and increased anxiety in the control group that was significant on the STAI, but not on the simple scale. This suggests that the impact depends on the design of the interventions and the sensitivity of the measurement tools.

There is also no consensus on the impact of audiovisual tools on satisfaction. In regard to informed consent, Glaser et al and Farrell et al did not identify an increase in satisfaction [16, 23], while Nehme et al did observe greater satisfaction when consent was supplemented with audiovisual tools [21], as in this study. Regarding overall satisfaction, Maghalian et al concluded that videos probably increase postoperative satisfaction in cesarean sections [14], while Kiernan et al found no improvement with digital technology in consent for surgeries [19]. These differences may be due to the variability in the audiovisual tools and measurement methods used, making direct comparisons difficult. In this case, there was a significant increase in satisfaction with the information and method of communication, highlighting the value of combining videos with face-to-face explanations. However, it had no impact on overall satisfaction, possibly due to contextual factors or individual expectations.

Following the completion of this study, the explanatory videos initially developed for research purposes have been incorporated into routine clinical practice at our institution. In addition, preprocedural consultations are increasingly being held before selected interventions, although their systematic integration into the institution’s daily practice is being progressively evaluated and adapted according to institutional capacities.

The study has limitations. The main limitation is not being able to determine the individual impact of the consultations and videos, as they were analyzed together. In addition, the information may be contaminated to a certain degree, as radiologists may have unintentionally enhanced the standard information. The wide range of procedures limits generalization, although it represents routine practice. Moreover, audiovisual materials have intrinsic limitations, as they may not be fully accessible to patients with language barriers or sensory disabilities. Future developments could include subtitles or translated versions for non-native Spanish speakers, as well as adapted formats such as audio descriptions or sign language interpretation to improve accessibility. Finally, the results may not be applicable to populations vulnerable to the digital divide, such as older adults.

This work is noteworthy because it is a randomized clinical trial that provides solid evidence to reinforce current guidelines aimed at promoting a more active clinical role of interventional radiologists [1, 10, 11, 25]. Until now, these recommendations lacked an empirical basis for this standard. Furthermore, this study demonstrates both the benefits of adopting these recommendations and the negative implications of ignoring them in healthcare practice. In conclusion, preprocedural consultations and explanatory videos have been shown to improve patients’ understanding, increase their satisfaction with the information and method of communication, and stabilize anxiety. Future studies should evaluate the benefits of extending this approach to all phases of longitudinal care, such as post-procedure consultations and hospital follow-up, analyzing its impact on quality, safety, and financial sustainability in order to consolidate a comprehensive model in modern IR.

## Supplementary information

ELECTRONIC SUPPLEMENTARY MATERIAL

## Abbreviation

IR: Interventional radiology
