# Supplementary material for: Impact of the clinical role of interventional radiologists: results of the CLINTERVENTIONAL randomized controlled trial
Source: Eur Radiol. 2025 Jun 17;35(12):7658–68. doi: 10.1007/s00330-025-11757-0 (PMC12634745; doi:10.1007/s00330-025-11757-0)
Supplement: Supplementary file 1 — ELECTRONIC SUPPLEMENTARY MATERIAL [file 330_2025_11757_MOESM1_ESM.pdf]

Impact of the clinical role of interventional radiologists: Results of the CLINTERVENTIONAL Randomized Controlled Trial

ELECTRONIC SUPPLEMENTARY MATERIAL

Appendix 1

Clinical Trial Information Sheet and Sample Informed Consent Form.

PATIENT INFORMATION SHEET

**Study Title**	Analysis of the impact of outpatient consultations and audiovisual tools on the patient experience in vascular interventional radiology
**Principal Investigator**	
**Site**	Reina Sofia University Hospital of Córdoba

Introduction

We are writing to inform you about a research study you are invited to participate in. The study has been submitted to, reviewed, and approved by the Research Ethics Committee of the Province of Córdoba.

Our intention is that you receive sufficient, correct information so that you are able to decide whether you agree to participate in this study or not. Please read this information sheet carefully and we will answer any questions you may have.

In addition, you may consult with any other people you deem appropriate.

Voluntary participation

We are inviting you to participate in the study because you have been asked to undergo a procedure that will be performed by the Vascular Interventional Radiology Department of the Reina Sofía University Hospital in Córdoba. You should know that your participation in this study is voluntary and that you may decide NOT to participate. If you decide to participate, you may change your decision and withdraw your consent at any time without this changing your relationship with your physician or negatively affecting your healthcare.

Study outcome measures

The main objective of the study is to determine if the implementation of outpatient consultations and the use of explanatory audiovisual tools before performing vascular interventional radiology procedures improve patients' understanding of the procedures, improve satisfaction with the information provided, and decrease anxiety caused by the procedure.

Study description

This study aims to include a total of 428 patients who will undergo procedures performed by Vascular Interventional Radiology Department.

Vascular interventional radiology is an area of medical practice focused on the diagnosis, treatment, and clinical management of patients using minimally invasive procedures that are guided and directed using imaging techniques. Vascular interventional radiology has developed rapidly since its inception at the end of the 20th century and has contributed to some of the most important medical innovations in recent times. However, this development has not been uniform. The expansion of technology and vascular interventional radiology techniques has not led to proportional clinical progress or an adequate development of communication skills.

This study aims to analyze and identify possible ways to improve communication with patients who will undergo vascular interventional radiology procedures. To this end, two ways of communicating information on vascular interventional radiology procedures will be compared:

- On the one hand, the usual way, in which information on the procedure is provided by the physician who orders it.
- On the other hand, an experimental form, in which the patient also has access to an explanatory video on the procedure and has a consultation with an interventional radiologist.

If you agree to participate in our study, you will be randomly assigned to a control group (in which you will be informed as usual) or to an experimental group (in which, in addition to being informed as usual, you will have access to a video explaining the procedure and will have a consultation with an interventional radiologist). You will have a 50% chance of entering either study group.

Study activities

The period you will participate in the study will last for approximately 14 days.

This study will begin when your physician requests a vascular interventional radiology procedure and communicates information regarding the procedure to you.

Your participation in this study will involve the following:

- First, we will contact you by telephone to invite you to participate in the study.
- Second, you will have a consultation in which you will be informed about the study and your questions about the study will be answered.
- Third, if you are assigned to the experimental group, you will have access to an explanatory video about the procedure and will have another consultation with an interventional radiologist, who will explain details about your procedure. If you are assigned to the control group, you will not have to attend this consultation.
- Fourth, you will answer a series of surveys that will allow us to evaluate the study's objectives. The surveys will be conducted after each consultation and on the day of the procedure (before and after the procedure).

In total, you will have to attend one consultation and complete three surveys if you are assigned to the control group or attend two consultations and complete four surveys if you are assigned to the experimental group.

A summary of the study activities is attached in the following table:

Procedures	Screening visit (Day 0)	Visit 2 (Day 1–7) <u>(experimental group only)</u>	Visit 3 (Day of the procedure, before it is performed)	Visit 4 (Day of the procedure, after it is performed)
Screening criteria	X			
Informed consent	X			
Randomization	X			
Consultation with an interventional radiologist and access to an educational video		X		
Survey/data collection	X	X	X	X

Risks and inconveniences arising from participation in the study

You will not be exposed to any further risks as a consequence of this study, as no extra tests will be performed and no additional medication will be administered.

Participation in the study may cause you some inconvenience, such as having to attend an outpatient consultation and having to answer some surveys or questionnaires.

As a study participant, you are responsible for completing all the study visits and activities.

Potential benefits

There are currently no clinical trials that have analyzed the usefulness of consultations and audiovisual aids in vascular interventional radiology. With your participation, you will have contributed to a trial on this topic, which is of great interest to help standardize clinical practice in vascular interventional radiology. What's more, if you are assigned to the experimental group, you will have access to an explanatory video about your procedure and will have a consultation with an interventional radiologist.

Personal data protection

The research team undertakes to comply with Organic Law 3/2018, on Personal Data Protection and Guarantee of Digital Rights.

The data collected for the study will be identified by a code, so no information that can identify you is included. Only your study doctor/collaborators will be able to link these data to you and your medical record. Therefore, your identity will not be disclosed to any individual except in case of a medical emergency or legal requirement. The processing, communication, and transfer of personal data of all participants shall be pursuant to the provisions of the law.

Access to your personally identifiable information will be restricted to the study doctor/collaborators, health authorities, and the Research Ethics Committee when required to verify personal data, clinical study procedures, and compliance with good clinical practice standards (always maintaining data confidentiality pursuant to current legislation).

The data will be collected in a research file that will be the institution's responsibility and will be processed within the framework of your participation in this study.

In accordance with the provisions of the data protection legislation, you may exercise your rights of access, rectification, opposition, and cancellation of the data. You can also limit the processing of data that are incorrect as well as request a copy or request that the data you have provided for the study be transferred to a third party (portability).

To exercise your rights, please contact the study's principal investigator. We remind you that the data cannot be deleted even if you stop participating in the trial. This is in order to ensure the validity of the research and to comply with legal duties and medication authorization requirements. You also have the right to contact the Data Protection Agency if you are not satisfied.

If you decide to withdraw your consent to participate in this study, no new data will be added to the database, but the data already collected will be used.

Expenses and financial compensation

Neither the investigators nor the site receive any financial compensation from the study.

Your participation in the study will not incur any additional costs to you.

Other important information

A description of this clinical trial will be available at https://clinicaltrials.gov.

You should be aware that you may be excluded from the study if the study investigators deem it appropriate either for safety reasons or because they believe that you are not complying with established procedures. In either case, you will receive an adequate explanation of the reason for your withdrawal from the study.

By signing the attached consent form, you agree to comply with the study procedures described to you.

As a study participant, if you stop coming to visits without withdrawing consent, the research team may follow up with you.

Contact in case of questions

If you have any questions or need further information during your participation, please contact Dr.____ of the ___________________________________ department at the telephone number___________________.

PARTICIPANT INFORMED CONSENT FORM

Study Title: Analysis of the impact of outpatient consultations and audiovisual tools on the patient experience in vascular interventional radiology.

I (participant's name and surname):______________________________________

- have read the information sheet about the study given to me.
- was able to ask questions about the study.
- have received enough information about the study.
- have spoken to (investigator's name):__________________________________
- understand that my participation is voluntary.
- understand that I can withdraw from the study:
  - Whenever I want.
  - Without having to give an explanation.
  - Without it impacting my medical care.

I wish to be informed of information arising from the research that may be relevant to my health:

YES ☐ NO ☐

I will receive a signed and dated copy of this informed consent document. I freely agree to participate in the study.

Participant's name: Researcher's name:

Date:______/______/______ Date:______/______/______

Participant's signature: Investigator's signature:

When IC is obtained in persons with a modified capacity to give their IC.

Name of the legal representative or relative: Researcher's name:

Date:______/______/______ Date:______/______/______

Legal representative's signature: Investigator's signature:

Appendix 2

Information on interventional procedures: Access to videos.

ENDOVASCULAR RECANALIZATION

What does it involve?

This is a procedure performed by interventional radiologists that allows for minimally invasive treatment of vessels (either arteries or veins) that have become blocked or have significant narrowing.

The procedure is performed under local anesthesia and through a small incision in the groin or arm. The vascular tree is accessed through this incision. Once inside the body, the medical team will navigate through the body's vessels under continuous X-ray monitoring and using contrast dye and different devices such as catheters until the diseased vessels are reached.

There are different alternatives for treating a diseased vessel, such as angioplasty or dilatation and placement of a stent or prosthesis in the area of the lesion in order to open it up and reestablish normal flow.

How will you benefit?

This procedure allows for correcting the obstruction or narrowing of a vessel, restoring flow, and improving blood supply to the affected area.

It is a minimally invasive procedure that can replace the need for surgery, which entails more risks and requires longer recovery and hospitalization times.

How will you prepare?

This procedure requires hospitalization, so you will usually be admitted in the afternoon the day before the procedure.

You must talk to your doctor about the medication you are taking because certain medications must be stopped or changed.

You must fast for 8 hours before the procedure. However, you may take your medication with a small amount of water.

What are the risks?

It is common to have discomfort or a bruise at the puncture site, which will go away in a few days.
Other possible complications include bleeding or blood clots at the puncture site or in other areas of the body. These complications are uncommon, but they may result in a prolongation of hospitalization and, exceptionally, they may be life-threatening. However, if any complications occur, please be assured that suitable measures will be taken to try to resolve them.

What can you expect after the procedure?

You must rest in bed for 24 hours.
If there are no incidents, you may be discharged the following day.

It is possible that you will have to take a medicine indefinitely.

PLACEMENT OF A TUNNELED CENTRAL VENOUS CATHETER

What does it involve?

This is a procedure performed by interventional radiologists under local anesthesia. It consists of placing a flexible plastic inside a vein that is usually in the neck but exceptionally may be in other areas of the body.

The medical team will use different radiological techniques, such as ultrasound and X-rays, to perform the procedure. The vein, which is usually in the neck, will be punctured with a small incision. Then, a tunnel will be made under the skin through which the catheter is placed so that one end of the catheter is inside a central vein or the heart and the other end is on the outside.

How will you benefit?

Depending on the catheter's purpose, it will be possible to perform dialysis, administer treatments, or perform analyses that are needed through the catheter without having to prick the patient's arm.

How will you prepare?

The procedure is usually performed on an outpatient basis, so no hospitalization is required and you can go home after the procedure.

You must talk to your doctor about the medication you are taking because certain medications must be stopped or changed.

You must fast for 8 hours before the procedure. However, you may take your medication with a small amount of water.

It is not recommendable to come in for the procedure alone or to drive after it, since medication that makes you relax may be used during the procedure.

What are the risks?

It is a safe procedure with a low probability of complications.

It is common to feel some pain or slight discomfort in the area, but these effects usually go away in a few days.

Other possible complications include infection, bleeding, or venous blood clots. These complications are uncommon, but they can lead to hospitalization and, very exceptionally, they can be life-threatening. However, if any complications occur, please be assured that suitable measures will be taken to try to resolve them.

What can you expect once the catheter has been placed?

The catheter can be used immediately after placement.

You must avoid getting the catheter and incisions wet.

The wounds must be treated every day until they are healed.

In the future, it may be necessary to exchange the catheter for a new one or remove it due to deterioration.

FISTULOGRAPHY AND ENDOVASCULAR REPAIR OF HEMODIALYSIS ARTERIOVENOUS FISTULAS

What does it involve?

The procedure is performed by interventional radiologists under local anesthesia. It allows for assessing the condition of your hemodialysis fistula. It also makes it possible to treat injuries that cause malfunction of the fistula.

The medical team will use radiological techniques, such as ultrasound and X-rays, and will use iodinated contrast dye during the procedure.

If there are lesions that cause malfunction of the fistula, such as stenosis or narrowing, they will be treated by angioplasty or dilation and by placing stents or prostheses to allow for adequate blood flow and to ensure good dialysis sessions.

How will you benefit?

This procedure allows for assessing the condition, function, and permeability of your hemodialysis fistula.

It also allows for identifying and treating lesions that cause malfunction of the fistula in the same procedure. This makes it possible to prolong the life and usefulness of your fistula as much as possible.

How will you prepare?

The procedure is usually performed on an outpatient basis, so no hospitalization is required and you can go home after the procedure.

You must talk to your doctor about the medication you are taking because certain medications must be stopped or changed.

You must fast for 8 hours before the procedure. However, you may take your medication with a small amount of water.

It is not recommendable to come in for the procedure alone or to drive after it, since medication that makes you relax may be used during the procedure.

What are the risks?

It is a safe procedure with a low probability of complications. It is common to have discomfort or a bruise at the puncture site, which will go away in a few days.

Other possible complications include infection, bleeding, or blood clots in the vessels studied. These complications are uncommon, but they can lead to hospitalization and, very exceptionally, they can be life-threatening. However, if any complications occur, please be assured that suitable measures will be taken to try to resolve them.

What can you expect after the procedure?

After your fistula has been checked and treated, you will be able to undergo dialysis again without the problems you had.

You must keep a bandage on your arm for a few hours.

You must avoid exertion with your arm for a few days.

ENDOVASCULAR EMBOLIZATION

What does it involve?

This is a procedure performed by interventional radiologists that allows for minimally invasive and very selective closure of diseased vessels (either arteries or veins).

The procedure is performed under local anesthesia and through a small incision in the groin or arm. The vascular tree is accessed through this incision. Once inside the body, the medical team will navigate through the body's vessels under continuous X-ray monitoring and using contrast dye and different devices such as catheters until the diseased vessels are reached.

There are different materials and substances to close the diseased vessels in order to stop blood circulation and solve the problem.

How will you benefit?

This procedure allows for closing or sealing a diseased vessel in any location of the body through a puncture in the groin or arm.

It is a minimally invasive procedure that can replace the need for surgery, which entails more risks and requires longer recovery and hospitalization times.

How will you prepare?

This procedure requires hospitalization, so you will usually be admitted in the afternoon the day before the procedure.

You must talk to your doctor about the medication you are taking because certain medications must be stopped or changed.

You must fast for 8 hours before the procedure. However, you may take your medication with a small amount of water.

What are the risks?

It is common to have discomfort or a bruise at the puncture site, which will go away in a few days.

You may have discomfort, fever, and pain that may last from 3 to 5 days and resolve with medical treatment.

Other possible complications include bleeding or blood clots at the puncture site or in other areas of the body. These complications are uncommon, but they may result in a prolongation of hospitalization and, exceptionally, they may be life-threatening. However, if any complications occur, please be assured that suitable measures will be taken to try to resolve them.

What can you expect after the procedure?

After the procedure, you must rest for 24 hours.

Discharge from the hospital will depend on the type of embolization performed and how you progress.

PERCUTANEOUS BIOPSY

What does it involve?

A percutaneous biopsy is a procedure performed by interventional radiologists under local anesthesia. It consists of extracting a small tissue sample (liver, kidney, bone, lymph node, etc.) for analysis. The medical team will use different radiological techniques, such as ultrasound or CT scan, to identify the area to be analyzed.

How will you benefit?

A diagnosis can be accurately and safely made with this procedure, making it unnecessary to perform a surgical biopsy, which is more invasive and requires longer recovery and hospitalization times.

The biopsy result will help your physician identify the cause and extent of your disease, make a diagnosis, and decide on a treatment plan.

How will you prepare?

Most biopsies require hospitalization, so you will usually be admitted in the afternoon the day before the procedure.

You must talk to your doctor about the medication you are taking because certain medications must be stopped or changed.

You must fast for 8 hours before the biopsy. However, you may take your medication with a small amount of water.

What are the risks?

It is a safe procedure with a low probability of complications.

It is common to have pain at the puncture site, which will go away in a few days.

Other possible complications include infection, internal bleeding, or puncture of a nearby organ. These complications are uncommon, but they may result in a prolongation of hospitalization and, very exceptionally, they may be life-threatening. However, if any complications occur, please be assured that suitable measures will be taken to try to resolve them.

What can you expect once the biopsy has been performed?

You must rest in bed for a few hours.

If there are no incidents, you will be discharged from the hospital after a few hours or the following day.

The sample will be sent for analysis by anatomic pathology specialists. The results will be communicated to you by your physician in a period of time that ranges from approximately one to two weeks.

PERCUTANEOUS DRAINAGE OF COLLECTIONS

What does it involve?

The procedure is performed by interventional radiologists under local anesthesia. It consists of placing a drainage catheter, which is a flexible plastic tube, into a collection.

The medical team uses radiological techniques such as ultrasound, X-rays, or CT scans to perform the procedure.

How will you benefit?

This procedure drains and empties an accumulation of fluid in a safe, minimally invasive, and effective manner. Generally, it makes it unnecessary to perform a more complex and invasive surgical procedure.

It will alleviate and improve the symptoms caused by the collection.

An analysis of the extracted fluid can help diagnose an infection and guide targeted treatment.

How will you prepare?

Most drainage procedures require hospitalization, so you will usually be admitted in the afternoon the day before the procedure.

You must talk to your doctor about the medication you are taking because certain medications must be stopped or changed.

You must fast for 8 hours before the drainage. However, you may take your medication with a small amount of water.

What are the risks?

It is a safe procedure with a low probability of complications.

It is common to have pain at the drainage site, which will go away in a few days.

Other possible complications include infection, internal bleeding, or puncture of a nearby organ. These complications are uncommon, but they may result in a prolongation of hospitalization and, very exceptionally, they may be life-threatening. However, if any complications occur, please be assured that suitable measures will be taken to try to resolve them.

What can you expect once drainage has been performed?

You must rest in bed for a few hours.

A series of care procedures will be performed in the days following the procedure, such as checking and flushing the catheter or changing the collection bag.
When the collection has resolved, the catheter will be removed quickly and easily.

PERCUTANEOUS BILIARY DRAINAGE

What does it involve?

The procedure is performed by interventional radiologists and is usually performed under local anesthesia and superficial sedation. It consists of placing a catheter, which is a flexible plastic tube, into the bile ducts through a small incision in the skin. This catheter will be connected to a collection bag that will collect the bile.

The medical team uses radiological techniques such as ultrasound and X-rays to perform the procedure.

How will you benefit?

This procedure allows for the bile produced in the liver to be emptied out of the body or into the intestine in an efficient and minimally invasive manner. This avoids problems resulting from an accumulation of bile, such as infection.

Emptying the bile should improve your condition and may alleviate symptoms resulting from its accumulation, such as itching or yellow skin.

Biliary drainage may also be necessary when preparing for surgery or other bile duct procedures such as a biopsy or prosthesis placement.

How will you prepare?

This procedure requires hospitalization, so you will usually be admitted in the afternoon the day before the procedure or you may already be hospitalized when the drainage is ordered.

You must talk to your doctor about the medication you are taking because certain medications must be stopped or changed.

You must fast for 8 hours before the drainage. However, you may take your medication with a small amount of water.

What are the risks?

It is common to have discomfort or a bruise at the puncture site, which will go away in a few days.

Other possible complications include infection, internal bleeding, or bile leaking into the abdominal or chest cavity. These complications are uncommon, but they may result in a prolongation of hospitalization and, exceptionally, they may be life-threatening. However, if any complications occur, please be assured that suitable measures will be taken to try to resolve them.

What can you expect once biliary drainage has been performed?

You must rest in bed for a few hours.

A series of care procedures will be performed in the days following the procedure, such as checking and flushing the catheter or changing the collection bag.

The length of time that a catheter remains in place varies and will depend on the reason why it was placed.

PERCUTANEOUS NEPHROSTOMY

What does it involve?

The procedure is performed by interventional radiologists under local anesthesia. It consists of placing a catheter, which is a flexible plastic tube, into the kidney through a small incision in the skin. This catheter will be connected to a collection bag that will collect the urine.

The medical team will use radiological techniques, such as ultrasound and X-rays.

How will you benefit?

This procedure allows for draining the urine produced in the kidney in an efficient and minimally invasive way. This ensures that the kidneys continue to function and avoids problems resulting from urine accumulation, such as infection.

It also allows for studying the urinary system through the administration of contrast dye.

How will you prepare?

A nephrostomy requires hospitalization, so you will usually be admitted in the afternoon the day before the procedure or you may already be hospitalized when your urologist orders it.

You must talk to your doctor about the medication you are taking because certain medications must be stopped or changed.

You must fast for 8 hours before the procedure. However, you may take your medication with a small amount of water.

What are the risks?

It is a safe procedure with a low probability of complications. It is common to have discomfort in the nephrostomy area, which will go away in a few days.

Other possible complications include infection, internal bleeding, or puncture of a nearby organ. These complications are uncommon, but they may result in a prolongation of hospitalization and, very exceptionally, they may be life-threatening. However, if any complications occur, please be assured that suitable measures will be taken to try to resolve them.

What can you expect after the procedure?

You must rest in bed for 24 hours.

A series of care procedures will be performed in the days following the procedure, such as checking and flushing the catheter or changing the collection bag.

The length of time that a catheter remains in place varies and will depend on the reason why it was placed.

Appendix 3

Specific questionnaires on knowledge and understanding of the procedures.

Scoring system: 1 point for each correct answer.

ENDOVASCULAR RECANALIZATION		
Patient code:	Telephone numbers:	VISIT ___
Date:		SCORE:
Who will perform the procedure?	O A surgeon	O An intensivist
	O A radiologist	O I have questions/I don't know
Is only a small incision necessary for the procedure?	O Yes	O I have questions/I don't know
	O No	
Will you be completely asleep for the procedure?	O Yes	O I have questions/I don't know
	O No	
Can a diseased vessel only be treated by placing a stent or prosthesis?	O Yes	O I have questions/I don't know
	O No	
Is it necessary to review the medication you are taking before the procedure?	O Yes	O I have questions/I don't know
	O No	
Is it necessary to fast before having the procedure?	O Yes, for 8 hours	O I have questions/I don't know
	O Yes, for 4 hours	
The procedure you are going to have performed, compared to a conventional surgical procedure	O Requires more hospitalization	O I have questions/I don't know
	O Is less invasive	
Is bleeding a possible complication?	O Yes	O I have questions/I don't know
	O No	
Are unwanted blood clots in a vessel a possible complication?	O Yes	O I have questions/I don't know
	O No	
Once the intervention has been completed	O It is necessary to rest for a few hours	O I have questions/I don't know
	O It is not necessary to rest	
Is it necessary to stay in the hospital for at least two days after the procedure?	O Yes	O I have questions/I don't know
	O No	
	O Yes	

Is it possible that you will have to take a medication for the rest of your life after the procedure?	O No	O I have questions/I don't know

TUNNELED CENTRAL VENOUS CATHETER PLACEMENT		
**Patient code:**	**Telephone numbers:**	**VISIT ___**
**Date:**		**SCORE:**
Who will place the catheter?	O A surgeon	O A nephrologist
	O A radiologist	O I have questions/I don't know
Are ultrasound and X-ray used to place the catheter?	O Yes<br>O No	O I have questions/I don't know
Will you be completely asleep for the procedure?	O Yes<br>O No	O I have questions/I don't know
How many incisions need to be made to place the catheter?	O 1<br>O 2	O I have questions/I don't know
Is it necessary to review the medication you are taking before the procedure?	O Yes<br>O No	O I have questions/I don't know
Is it advisable to come in for the procedure alone?	O Yes<br>O No	O I have questions/I don't know
Does its use depend on the catheter's purpose and can it be used for dialysis or analysis?	O Yes<br>O No	O I have questions/I don't know
Is bleeding a possible complication?	O Yes<br>O No	O I have questions/I don't know
Are venous blood clots a possible complication?	O Yes<br>O No	O I have questions/I don't know
Once the catheter is in place, do you to have to wait a week before you can use it?	O Yes<br>O No	O I have questions/I don't know
After catheter placement	O It is important that it doesn't get wet<br>O It doesn't matter if the catheter gets wet	O I have questions/I don't know
Is this catheter permanent for the rest of your life?	O Yes<br>O No, replacements may be necessary	O I have questions/I don't know

FISTULOGRAPHY AND HEMODIALYSIS FISTULA REPAIR				
**Patient code:**	**Telephone numbers:**		**VISIT ___**	
**Date:**			**SCORE:**	
Who will perform the procedure?	O	A surgeon	O	A nephrologist
	O	A radiologist	O	I have questions/I don't know
Are ultrasound and X-rays used during the procedure?	O	Yes	O	I have questions/I don't know
	O	No		
Will you be put completely to sleep?	O	Yes	O	I have questions/I don't know
	O	No		
If there are lesions that cause malfunction of the fistula, can they be treated in the same procedure?	O	Yes	O	I have questions/I don't know
	O	No		
Is it necessary to review the medication you are taking before the procedure?	O	Yes	O	I have questions/I don't know
	O	No		
Is it advisable to come in for the procedure alone?	O	Yes	O	I have questions/I don't know
	O	No		
Does this procedure prolong the life and usefulness of the fistula as much as possible?	O	Yes	O	I have questions/I don't know
	O	No		
Is bleeding a possible complication?	O	Yes	O	I have questions/I don't know
	O	No		
Are venous blood clots a possible complication?	O	Yes	O	I have questions/I don't know
	O	No		
Is it necessary to wait for a day after the procedure before dialysis?	O	Yes	O	I have questions/I don't know
	O	No		
Will you have to keep a pressure bandage on your arm when you finish?	O	Yes	O	I have questions/I don't know
	O	No		
After the procedure, will you have to avoid exerting your arm for a few days?	O	Yes	O	I have questions/I don't know
	O	No		

ENDOVASCULAR EMBOLIZATION		
**Patient code:**	**Telephone numbers:**	**VISIT ___**
**Date:**		**SCORE:**
Who will perform the procedure?	O A surgeon	O An intensivist
	O A radiologist	O I have questions/I don’t know
Is only a small incision necessary for the procedure?	O Yes	O I have questions/I don’t know
	O No	
Will you be completely asleep for the procedure?	O Yes	O I have questions/I don’t know
	O No	
Are there different materials and substances to close or seal diseased vessels?	O Yes	O I have questions/I don’t know
	O No	
Is it necessary to review the medication you are taking before the procedure?	O Yes	O I have questions/I don’t know
	O No	
Is it necessary to fast before having the procedure?	O Yes, for 8 hours	O I have questions/I don’t know
	O Yes, for 4 hours	
The procedure you are going to have performed, compared to a conventional surgical procedure	O Requires a longer recovery time	O I have questions/I don’t know
	O Is less invasive	
Is bleeding a possible complication?	O Yes	O I have questions/I don’t know
	O No	
Are unwanted blood clots in a vessel a possible complication?	O Yes	O I have questions/I don’t know
	O No	
Once the intervention has been completed	O It is necessary to rest for a few hours	O I have questions/I don’t know
	O It is not necessary to rest	
Is it necessary to stay in the hospital for at least two days after the procedure?	O Yes	O I have questions/I don’t know
	O No	
Is it possible you may have discomfort or fever in the days following the embolization?	O Yes	O I have questions/I don’t know
	O No	

PERCUTANEOUS BIOPSY				
**Patient code:**	**Telephone numbers:**		**VISIT ___**	
**Date:**			**SCORE:**	
Who will perform the biopsy?	O	A surgeon	O	An oncologist
	O	A radiologist	O	I have questions/I don't know
Will the biopsy be performed with ultrasound or CT control?	O	Yes	O	I have questions/I don't know
	O	No		
Will you be fully asleep for the biopsy?	O	Yes	O	I have questions/I don't know
	O	No		
Is it performed with a fine needle through a small skin incision?	O	Yes	O	I have questions/I don't know
	O	No		
Is it necessary to review the medication you are taking before the biopsy?	O	Yes	O	I have questions/I don't know
	O	No		
Is it necessary to fast before having the biopsy?	O	Yes, for 8 hours	O	I have questions/I don't know
	O	Yes, for 4 hours		
The biopsy you will undergo, compared to the surgical biopsy	O	Requires more hospitalization	O	I have questions/I don't know
	O	Is less invasive		
Is bleeding a possible complication of the biopsy?	O	Yes	O	I have questions/I don't know
	O	No		
Is puncture of nearby organs a possible complication of biopsy?	O	Yes	O	I have questions/I don't know
	O	No		
Once the biopsy has been performed	O	I will have to rest in bed for a few hours	O	I have questions/I don't know
	O	I will be able to go home		
Who analyzes the biopsy sample?	O	Anatomic pathology specialists	O	I have questions/I don't know
	O	Oncology specialists		
How long does it usually take to get the results?	O	1–2 weeks	O	I have questions/I don't know
	O	3–4 weeks		

PERCUTANEOUS DRAINAGE OF COLLECTIONS		
**Patient code:**	**Telephone numbers:**	**VISIT ___**
**Date:**		**SCORE:**
Who will perform the drainage?	O A surgeon	O An intensivist
	O A radiologist	O I have questions/I don't know
Are ultrasound, X-ray, or CT used to perform the drainage?	O Yes<br>O No	O I have questions/I don't know
Will you be completely asleep for the procedure?	O Yes<br>O No	O I have questions/I don't know
Will a bag be placed to collect the content of the collection?	O Yes<br>O No	O I have questions/I don't know
Is it necessary to review the medication you are taking before the procedure?	O Yes<br>O No	O I have questions/I don't know
Is it necessary to fast before having the drainage?	O Yes, for 8 hours<br>O Yes, for 4 hours	O I have questions/I don't know
Is one of the benefits of this intervention the ability to analyze the content of the collection?	O Yes<br>O No	O I have questions/I don't know
Is bleeding a possible complication?	O Yes<br>O No	O I have questions/I don't know
Is puncture of nearby organs a possible complication?	O Yes<br>O No	O I have questions/I don't know
Once the drainage catheter is placed	O It is necessary to rest for a few hours<br>O It is not necessary to rest	O I have questions/I don't know
Is it necessary to check and flush the drainage catheter after placing it?	O Yes<br>O No	O I have questions/I don't know
Should the drainage catheter remain in place for at least one month?	O Yes<br>O No	O I have questions/I don't know

PERCUTANEOUS BILIARY DRAINAGE		
**Patient code:**	**Telephone numbers:**	**VISIT ___**
**Date:**		**SCORE:**
Who will perform the procedure?	O A surgeon	O An intensivist
	O A radiologist	O I have questions/I don't know
Are ultrasound and X-ray used to perform biliary drainage?	O Yes	O I have questions/I don't know
	O No	
Does this procedure only allow for bile to be drained out of the body?	O Yes	O I have questions/I don't know
	O No	
Will a bag be placed to collect the bile?	O Yes	O I have questions/I don't know
	O No	
Is it necessary to review the medication you are taking before the procedure?	O Yes	O I have questions/I don't know
	O No	
Is it necessary to fast before having the biliary drainage?	O Yes, for 8 hours	O I have questions/I don't know
	O Yes, for 4 hours	
Is one of the benefits of this intervention to avoid having problems resulting from bile accumulation, such as infection?	O Yes	O I have questions/I don't know
	O No	
Is bleeding a possible complication?	O Yes	O I have questions/I don't know
	O No	
Is bile outflow into the abdominal cavity a possible complication?	O Yes	O I have questions/I don't know
	O No	
After placement of the biliary drainage catheter	O It is necessary to rest for a few hours	O I have questions/I don't know
	O It is not necessary to rest	
Is it necessary to check and flush the catheter after placement?	O Yes	O I have questions/I don't know
	O No	
Should the drainage catheter remain in place for at least one month?	O Yes	O I have questions/I don't know
	O No	

PERCUTANEOUS NEPHROSTOMY		
**Patient code:**	**Telephone numbers:**	**VISIT ___**
**Date:**		**SCORE:**
Who will perform the nephrostomy?	O A urologist	O A surgeon
	O A radiologist	O I have questions/I don't know
Are ultrasound and X-ray used to perform nephrostomy?	O Yes	O I have questions/I don't know
	O No	
Will you be completely asleep for the procedure?	O Yes	O I have questions/I don't know
	O No	
Will a bag be placed to collect urine?	O Yes	O I have questions/I don't know
	O No	
Is it necessary to review the medication you are taking before the procedure?	O Yes	O I have questions/I don't know
	O No	
Is it necessary to fast before having the nephrostomy?	O Yes, for 8 hours	O I have questions/I don't know
	O Yes, for 4 hours	
Does this procedure allow for draining urine produced in the kidney in a minimally invasive way?	O Yes	O I have questions/I don't know
	O No	
Is bleeding a possible complication?	O Yes	O I have questions/I don't know
	O No	
Is puncture of nearby organs a possible complication?	O Yes	O I have questions/I don't know
	O No	
After placement of the nephrostomy catheter	O It is necessary to rest for a few hours	O I have questions/I don't know
	O It is not necessary to rest	
Is it necessary to check and flush the nephrostomy catheter after placing it?	O Yes	O I have questions/I don't know
	O No	
Should the nephrostomy catheter remain in place for at least one month?	O Yes	O I have questions/I don't know
	O No	
